# Precision Glaucoma Surgery: A Mechanism-Based Review of MIGS and Contemporary Surgical Pathways

**DOI:** 10.3390/jcm15145461

**Published:** 2026-07-13

**Authors:** Wojciech Luboń, Adrian Smędowski, Mariola Dorecka

**Affiliations:** 1 Department of Ophthalmology, Faculty of Medical Sciences, Medical University of Silesia, 40-514 Katowice, Poland; mdorecka@sum.edu.pl; 2 Department of Ophthalmology, Professor K. Gibiński University Clinical Center, Medical University of Silesia, 40-514 Katowice, Poland; 3 Department of Pediatric Ophthalmology, Faculty of Medical Sciences, Medical University of Silesia, 40-514 Katowice, Poland; asmedowski@sum.edu.pl; 4 Department of Pediatric Ophthalmology, Professor K. Gibiński University Clinical Center, Medical University of Silesia, 40-514 Katowice, Poland; 5 GlaucoTech Co., 40-282 Katowice, Poland

**Keywords:** glaucoma surgery, microinvasive glaucoma surgery, MIGS, precision glaucoma surgery, Schlemm’s canal, Hydrus Microstent, trabecular bypass, aqueous humor outflow, intraocular pressure, trabeculectomy

## Abstract

Glaucoma surgery is entering a precision-based era in which surgical success is determined not only by intraocular pressure reduction, but also by the anatomical mechanism targeted, the expected durability of effect, safety profile, medication burden, quality of life, and preservation of future therapeutic options. Microinvasive glaucoma surgery (MIGS) has become an important component of this paradigm by expanding the surgical continuum toward earlier, anatomically targeted, and tissue-sparing intervention. However, MIGS does not represent a homogeneous procedural category; rather, it encompasses distinct approaches that modulate different components of aqueous humor dynamics, including the trabecular meshwork, Schlemm’s canal, distal collector channels, the subconjunctival space, the suprachoroidal pathway, and aqueous production. This narrative review synthesizes contemporary evidence and proposes a mechanism-based framework for glaucoma surgery, with particular emphasis on trabecular and Schlemm’s canal-based procedures as representative examples of physiologically oriented conventional outflow enhancement. Canal-based interventions, trabecular bypass, goniotomy, trabeculotomy, and canaloplasty are most appropriate when enhancement of conventional outflow can achieve clinically meaningful pressure and medication reduction, particularly in mild-to-moderate open-angle glaucoma. By contrast, subconjunctival filtration, trabeculectomy, and aqueous shunt surgery remain indispensable when lower target pressures are required, especially in advanced or rapidly progressive disease. Lens extraction and ciliary body-directed procedures further illustrate the importance of aligning surgical strategy with disease phenotype and anatomical mechanism. The future of glaucoma surgery will therefore depend on individualized procedure selection guided by glaucoma stage, target pressure, angle and lens anatomy, outflow pathway integrity, safety considerations, and long-term surgical sequencing.

## 1. Introduction

Glaucoma surgery is undergoing a profound conceptual reorientation, reflecting both the expanding evidence base for microinvasive procedures and the continuing role of established filtration and tube-based operations [1,2,3,4,5]. For decades, surgical intervention was largely regarded as a late-stage therapeutic option, typically reserved for eyes in which topical medication, laser therapy, or both had failed to achieve adequate intraocular pressure (IOP) control [2,3,4]. Within this traditional paradigm, trabeculectomy and aqueous shunt implantation constituted the principal surgical strategies, particularly in advanced disease requiring low target IOP [4,6,7]. These procedures remain indispensable in contemporary glaucoma care; however, their pressure-lowering efficacy is counterbalanced by intensive postoperative management and by the risk of hypotony, bleb-related infection, subconjunctival fibrosis, tube-related complications, and other potentially vision-threatening adverse events [4,6,7,8]. The emergence of microinvasive glaucoma surgery (MIGS), together with renewed interest in canal-based, subconjunctival, cyclophotocoagulation-based, and lens-related mechanisms of IOP modulation, has expanded the surgical continuum and challenged the historical separation between conservative treatment and late incisional surgery [1,2,3,4,5,9,10,11,12].

This evolution parallels a broader refinement in the understanding of glaucoma itself. Glaucoma is not a uniform pressure-mediated disorder, but a chronic, progressive, and biologically heterogeneous optic neuropathy in which IOP remains the only consistently modifiable therapeutic factor [1,2,3]. Although IOP lowering remains the central objective of treatment, modern surgical decision-making increasingly recognizes that IOP is not merely a numerical endpoint, but a surrogate expression of aqueous humor dynamics, anatomical configuration, and outflow resistance [10,11]. Rather, it reflects a complex and anatomically distributed system involving the trabecular meshwork, Schlemm’s canal, collector channels, episcleral venous pressure, the uveoscleral pathway, the ciliary body, lens-related anterior segment configuration, and, in filtration procedures, conjunctival and subconjunctival wound-healing responses [1,2,3,4,5,7,12]. Consequently, surgical success should be defined not only by the magnitude of pressure reduction, but also by durability, safety, medication reduction, quality of life, preservation of future surgical options, and the appropriateness of the anatomical target selected for the individual eye [2,3,4,13].

MIGS has become a central component of this precision-oriented paradigm by expanding the surgical spectrum toward earlier, anatomically targeted, and conjunctiva-sparing intervention [1,2,3,4]. However, MIGS should not be interpreted as a single operation, a uniform device category, or a simplified alternative to conventional glaucoma surgery [3,4,5]. It encompasses a heterogeneous spectrum of interventions that differ substantially in anatomical target, mechanism of action, efficacy, risk profile, postoperative burden, and long-term role within surgical sequencing [1,4,5]. Trabecular micro-bypass implants, Schlemm’s canal scaffolds, excisional or ablative goniotomy, ab interno trabeculotomy, canaloplasty-based procedures, suprachoroidal approaches, subconjunctival microshunts, and ciliary body-directed interventions all aim to reduce IOP, but they do so by modifying distinct components of aqueous humor dynamics [14,15,16,17,18,19,20,21,22,23,24,25,26,27,28,29,30,31,32,33,34,35]. Therefore, grouping these procedures under a single procedural label may obscure clinically meaningful differences in expected pressure reduction, safety profile, postoperative monitoring, and future surgical sequencing [2,3,4,5,13]. A contemporary interpretation of glaucoma surgery requires a mechanism-based classification that reflects anatomical target and pressure-lowering pathway rather than device type or incision size alone [1,2,3,4,5].

Among contemporary MIGS approaches, trabecular and Schlemm’s canal-based procedures provide a clinically important example of mechanism-directed intervention within the conventional outflow pathway [1,14,15,17]. By reducing resistance at the level of the trabecular meshwork, enhancing access to Schlemm’s canal, or engaging a broader segment of the canalicular system, these procedures aim to improve physiological aqueous drainage while preserving the conjunctiva for potential future filtration surgery [14,15,35,36,37,38]. Their clinical value is greatest when moderate IOP reduction and reduction in medication burden are sufficient therapeutic goals, particularly in patients with mild-to-moderate open-angle glaucoma undergoing cataract surgery [9,33,34,35]. However, because their efficacy remains dependent on distal outflow function and constrained by episcleral venous pressure, they should be positioned within a broader surgical strategy in which subconjunctival filtration, trabeculectomy, and aqueous shunt implantation remain necessary when substantially lower target pressures are required [1,6,7,26].

This heterogeneity has direct implications for clinical decision-making and surgical sequencing [1,4]. The central question in contemporary glaucoma surgery is no longer whether a procedure is minimally invasive, nor whether one device is universally superior to another, but which anatomical pathway should be targeted in a specific eye, at a specific stage of disease, to achieve an IOP level compatible with long-term optic nerve preservation while minimizing treatment burden and surgical risk [1,2,3,4,5]. In mild-to-moderate open-angle glaucoma, particularly in patients undergoing cataract surgery, trabecular and Schlemm’s canal-based MIGS may reduce medication dependence and provide additional IOP lowering with a favorable safety profile [13,15,36,37,38]. In angle-closure disease, lens extraction may directly address the anatomical mechanism of angle narrowing by deepening the anterior chamber and widening the iridocorneal angle and should therefore be regarded as a pathophysiologically meaningful intervention rather than merely a visually rehabilitative procedure [10,11]. In advanced, refractory, or rapidly progressive glaucoma, however, trabeculectomy and aqueous shunt surgery remain essential when lower target pressures are required beyond the expected capacity of conventional outflow enhancement [4,7].

Precision glaucoma surgery therefore depends on aligning the intervention with disease phenotype, anatomical mechanism, target IOP, safety requirements, and long-term therapeutic sequencing [1,4]. In practical terms, precision glaucoma surgery may be defined as the individualized selection and sequencing of surgical interventions based on the integration of disease severity, rate of progression, target and baseline IOP, angle and lens anatomy, ocular surface status, conjunctival reserve, prior surgical history, medication tolerance, and the anticipated site of aqueous outflow resistance [2,3,4,5]. Advances in anterior segment imaging, assessment of aqueous outflow function, biomaterials, sustained drug delivery, surgical navigation, and predictive analytics may further support the transition from standardized stepwise algorithms toward individualized surgical planning [39,40]. In this framework, MIGS should be understood as a group of mechanism-specific procedures that complement, rather than replace, established filtration and tube-based surgery when selected according to anatomical target, expected pressure-lowering capacity, and long-term therapeutic need [6,7].

Accordingly, this narrative review examines contemporary glaucoma surgery through a pathophysiology-driven and mechanism-based framework. Particular emphasis is placed on trabecular and Schlemm’s canal-based procedures as representative examples of physiologically oriented conventional outflow enhancement, while situating these techniques within the wider context of subconjunctival filtration, suprachoroidal surgery, cyclophotocoagulation-based strategies, lens-related anatomical modulation, and conventional filtering procedures [14,15,16,32,33,34]. The objective is not to present MIGS as a collection of competing devices, but to define its role within precision glaucoma surgery, in which the optimal procedure is determined by disease mechanism, anatomical target, required IOP reduction, long-term safety, and preservation of future therapeutic flexibility.

## 2. Methods of Literature Identification and Evidence Synthesis

This narrative review was based on a structured search of PubMed/MEDLINE, Scopus, and the Cochrane Library for English-language publications addressing contemporary glaucoma surgery, including MIGS, filtration procedures, aqueous shunts, cyclodestructive techniques, and lens-based surgical interventions. Priority was given to randomized controlled trials, systematic reviews, meta-analyses, Cochrane reviews, and landmark studies with direct clinical relevance. Evidence was evaluated qualitatively according to study design, methodological quality, duration of follow-up, consistency of findings, and relevance to surgical decision-making. Given the heterogeneity of procedures, study designs, and outcome measures, the evidence was synthesized narratively to support a mechanism-based framework for precision glaucoma surgery.

## 3. Pathophysiological and Mechanism-Based Framework for Contemporary Glaucoma Surgery

The evolution of glaucoma surgery is inseparably linked to a more refined understanding of aqueous humor dynamics, glaucoma heterogeneity, and the anatomical diversity of pressure-generating mechanisms [1,2,3,4,5]. Although IOP remains the only consistently modifiable therapeutic factor, the mechanisms responsible for IOP elevation differ substantially across glaucoma subtypes and even between individual eyes with the same clinical diagnosis [3,4,5]. Surgical planning should therefore extend beyond the selection of a procedure capable of lowering pressure and should instead consider the anatomical pathway through which pressure reduction is most likely to be achieved [1,4]. A precision-oriented approach requires identification of the relevant outflow or aqueous-production mechanism and selection of the procedure that offers the most favorable balance between efficacy, safety, durability, and preservation of future therapeutic options [5,13].

In primary open-angle glaucoma, the conventional outflow pathway remains the principal anatomical target of many contemporary surgical procedures [15,17]. Increased resistance at the level of the trabecular meshwork and the inner wall of Schlemm’s canal provides the rationale for trabecular micro-bypass implantation, Schlemm’s canal scaffolding, trabecular ablation, excisional goniotomy, ab interno trabeculotomy, and canaloplasty-based surgery [17,18,19,20,27,28,29,30,31,35]. These interventions share a common physiological objective: to restore or enhance aqueous drainage through the eye’s native outflow system rather than create an external filtration route [32,33,34]. However, the conventional outflow pathway is not a uniform circumferential conduit. Outflow is segmental, and the functional contribution of individual collector channel regions may vary between eyes [35]. This anatomical variability explains why focal trabecular bypass may be effective in selected cases, whereas broader canal-directed procedures, including segmental Schlemm’s canal scaffolding, ab interno trabeculotomy, and canaloplasty, may increase the probability of recruiting functional distal outflow pathways [15,17,24,30,31,32,33,35,36,37,38].

The pressure-lowering potential of trabecular and Schlemm’s canal-based surgery is nevertheless constrained by distal outflow resistance [1,2,3,4,35]. If the dominant site of resistance lies beyond Schlemm’s canal, within collector channels, aqueous veins, or episcleral venous drainage, bypassing or removing trabecular tissue may produce only limited IOP reduction [1,2,3]. In addition, episcleral venous pressure imposes a physiological lower boundary on the IOP that can be achieved through conventional outflow enhancement [2,3]. This therapeutic ceiling is central to patient selection. Canal-based and trabecular procedures are most appropriate when moderate IOP reduction and medication reduction are clinically sufficient [27,28,29,30,31,32,33,34,35], whereas filtration procedures and aqueous shunt implantation are generally required when low-teen or single-digit target IOPs are necessary, particularly in advanced or rapidly progressive disease [4,6,7].

Other surgical pathways are based on distinct pathophysiological principles and should be interpreted according to their anatomical target rather than grouped solely by invasiveness [1,2,3,4,5]. Suprachoroidal and supraciliary procedures aim to enhance uveoscleral outflow through an ab interno approach that is independent of the conventional outflow pathway [16]. By utilizing the pressure gradient between the anterior chamber and the suprachoroidal space, these procedures provide an alternative mechanism of IOP reduction that does not rely on trabecular, Schlemm’s canal, or distal collector channel function. This strategy is conceptually attractive in eyes where conventional outflow resistance may limit the effectiveness of canal-based interventions. However, clinical experience with suprachoroidal devices has highlighted the importance of long-term safety assessment, particularly regarding endothelial preservation and device-related complications [13,16]. Subconjunctival filtration procedures, including XEN implantation and trabeculectomy, create an alternative drainage route from the anterior chamber to the subconjunctival space [4,6,21,22,23,26]. This strategy may achieve greater pressure reduction than conventional outflow MIGS, but surgical success becomes dependent on conjunctival wound healing, bleb morphology, fibrosis modulation, antimetabolite use, and postoperative intervention burden [21,22,23,26]. Ciliary body-directed procedures act through a different mechanism by reducing aqueous humor production and include endoscopic cyclophotocoagulation (ECP), conventional transscleral cyclophotocoagulation (CW-TSCPC), and micropulse transscleral cyclophotocoagulation (MP-TSCPC) [12,25]. ECP enables direct endoscopic treatment of the ciliary processes, whereas transscleral techniques deliver laser energy without intraocular access. Compared with conventional CW-TSCPC, micropulse treatment applies laser energy in short intermittent pulses, reducing collateral thermal damage while maintaining effective IOP reduction in appropriately selected eyes [12,25].

Lens-related anterior segment anatomy constitutes an additional and clinically important mechanism of IOP modulation, particularly in angle-closure disease [10,11]. Lens extraction can deepen the anterior chamber, widen the iridocorneal angle, reduce pupillary block, and relieve lens-related crowding [11]. In this setting, cataract extraction or clear-lens extraction should be regarded not only as a visually rehabilitative procedure, but also as a pathophysiologically targeted intervention directed at the anatomical mechanism of angle closure [10,11]. In open-angle glaucoma, phacoemulsification may produce modest IOP reduction and provides a practical platform for combined MIGS; however, the incremental effect of the glaucoma procedure should be interpreted separately from the pressure-lowering effect of cataract surgery itself [9,25,26,36,37,38].

A mechanism-based classification is therefore more clinically informative than a purely device-based or incision-based taxonomy [1,2,3,4,5]. Trabecular and Schlemm’s canal-based procedures are most appropriate when enhancement of conventional outflow is expected to achieve the required target IOP [27,28,29,30,31,32,33,34,35]. Suprachoroidal approaches seek to augment uveoscleral drainage but require durable safety validation [13,16]. Subconjunctival microshunts and conventional filtration surgery are more suitable when greater pressure reduction is required, although they introduce bleb-related biology and a greater postoperative management burden [21,22,23,26]. Ciliary body-directed procedures reduce aqueous production and may be considered in selected eyes or combined strategies [12,25], whereas lens extraction is central when lens-related anterior segment configuration contributes to IOP elevation [10,11].

This framework provides the conceptual foundation for precision glaucoma surgery [2,3,4,5]. The optimal operation is not necessarily the newest, least invasive, or most technologically advanced intervention, but the procedure whose anatomical target, pressure-lowering capacity, safety profile, and durability best correspond to the patient’s disease mechanism, glaucoma stage, target IOP, ocular anatomy, and long-term therapeutic needs [2,13]. In this model, MIGS is not a peripheral category of less invasive procedures, but an integral component of precision glaucoma surgery when selected according to mechanism, anatomy, and clinical objective [3,4,13]. A mechanism-based overview of the principal surgical pathways, their anatomical targets, pressure-lowering mechanisms, clinical roles, and key limitations is summarized in Table 1.

## 4. Canal-Based and Trabecular Surgery: From Focal Bypass to Segmental Outflow Restoration

Trabecular and Schlemm’s canal-based surgery represents one of the most important developments in contemporary glaucoma management because it targets the conventional outflow pathway without creating a subconjunctival filtration bleb [15,17,18,19,20,27,28,29,30,31,32,33,34,35]. After defining the broader mechanism-based framework, this section focuses on procedures designed to reduce resistance at the level of the trabecular meshwork, the inner wall of Schlemm’s canal, and proximal access to collector channels [17,18,19,20]. Their shared objective is to enhance physiological aqueous humor drainage while preserving conjunctival integrity, reducing medication burden, and avoiding the wound-healing dependence associated with bleb-forming surgery [27,28,29,30,31,32,33,34,35]. Accordingly, these procedures should be understood not as substitutes for trabeculectomy or aqueous shunt implantation in eyes requiring very low target IOP, but as anatomically targeted, tissue-sparing interventions for patients in whom moderate pressure reduction is clinically appropriate [2,3,4,6,7].

Focal trabecular micro-bypass surgery is the most localized form of conventional outflow enhancement [14,27,34]. The iStent family of devices represents the prototypical approach in this category, creating a direct communication between the anterior chamber and Schlemm’s canal through a limited trabecular bypass [14,27,34]. Clinical evidence and systematic reviews indicate that trabecular micro-bypass implantation, particularly when combined with phacoemulsification, can reduce IOP and medication burden in selected patients with mild-to-moderate open-angle glaucoma [27,34]. However, the focal nature of the intervention is also its principal limitation. If the stent is positioned away from a functional collector channel region, or if resistance is predominantly distal to Schlemm’s canal, the magnitude of pressure reduction may be modest [1,2,3,4,14,27,34]. Thus, focal bypass procedures are most rational when the clinical objective is medication reduction or moderate IOP lowering rather than achievement of very low target pressure [27,34].

The Hydrus Microstent extends the focal bypass concept by combining trabecular bypass with segmental scaffolding of Schlemm’s canal [15,17,32,33,36,37,38]. Implanted ab interno through the trabecular meshwork, the device spans approximately three clock hours of Schlemm’s canal and is designed to maintain canal patency over a broader segment, thereby potentially improving access to multiple collector channel ostia [17,36,37,38]. In this respect, Hydrus is best interpreted as a canal-modulating scaffold rather than as a purely focal trabecular bypass implant [33,36,37,38]. Randomized trials evaluating Hydrus implantation with phacoemulsification demonstrated additional IOP-lowering and medication-sparing benefit compared with cataract surgery alone, while three-year and long-term HORIZON data supported durability beyond the early postoperative period [15,36,37,38]. Cochrane evidence, network meta-analysis, and direct comparative data, including the COMPARE trial, further position Hydrus among the more extensively evaluated Schlemm’s canal-based MIGS procedures [17,32,33]. The clinical role of Hydrus should nevertheless remain mechanism-specific rather than device-centered. Its strongest evidence relates to patients with mild-to-moderate open-angle glaucoma, particularly when cataract surgery is already planned and when the therapeutic goals include moderate IOP reduction, reduction in topical medication burden, preservation of the conjunctiva, and maintenance of future surgical options [15,32,33,36,37,38]. Like all conventional outflow procedures, its efficacy depends on the functional integrity of distal outflow pathways and remains constrained by episcleral venous pressure [1,2,3,4]. Therefore, Hydrus should not be framed as a universal alternative to trabeculectomy or aqueous shunt surgery, but as a representative example of precision MIGS when Schlemm’s canal modulation matches the patient’s anatomical and therapeutic profile [1,2,3,4,6,7].

Implant-free trabecular procedures provide another strategy for enhancing conventional outflow while avoiding permanent device implantation [18,19,20,28,29]. Trabectome surgery ablates a portion of the trabecular meshwork and inner wall of Schlemm’s canal, whereas Kahook Dual-Blade goniotomy excises a strip of trabecular tissue to reduce proximal outflow resistance [18,19,20,28,29]. These approaches may be particularly attractive when avoidance of intraocular implant material is desirable, when a broader trabecular segment is targeted, or when cost and device availability influence surgical choice [28,29,30,31,35,39]. Available clinical evidence suggests that trabecular ablation and excisional goniotomy can reduce IOP and medication burden, especially in mild-to-moderate glaucoma and when combined with phacoemulsification [18,19,20,28,29]. Transient hyphema is a common and mechanistically expected postoperative finding, reflecting direct communication between the anterior chamber and Schlemm’s canal after trabecular tissue disruption [18,19,20,29,30,31].

Ab interno trabeculotomy using microhook techniques further highlights the importance of treatment extent [30,31]. By incising the trabecular meshwork and inner wall of Schlemm’s canal from within the anterior chamber, microhook trabeculotomy allows the surgeon to modulate the circumferential range of conventional outflow access [30,31]. Studies comparing different extents of trabeculotomy suggest that the treated arc may influence both efficacy and safety, reinforcing a central question for canal-based surgery: how much of the trabecular–canalicular system should be opened or modified to achieve durable pressure reduction without unnecessary tissue disruption [30,31]? This concept aligns with the rationale of segmental canal scaffolding and canaloplasty, both of which move beyond a strictly focal intervention toward broader engagement of Schlemm’s canal and distal outflow pathways [24,30,31,32,33,35,36,37,38].

Canaloplasty-based procedures represent a complementary approach to conventional outflow restoration [24,35]. Rather than bypassing or excising trabecular tissue alone, canaloplasty aims to dilate Schlemm’s canal and improve access to the distal collector channel system [24,35]. Unlike subconjunctival filtration surgery, it does not depend on bleb formation; unlike focal trabecular stenting, it seeks to influence a broader segment of the canal [4,24,35]. The STREAMLINE randomized controlled trial comparing canaloplasty with trabecular micro-bypass stent implantation reflects growing interest in procedures that address Schlemm’s canal beyond a single focal access point [35]. Similarly, studies combining phacoemulsification with viscocanalostomy have evaluated not only IOP reduction but also changes in anterior segment anatomy and ocular biomechanical parameters, suggesting that canal-based surgery may influence aqueous outflow dynamics and anterior segment physiology in ways not fully captured by postoperative IOP alone [24].

Overall, trabecular and Schlemm’s canal-based procedures occupy a distinct and clinically important position within precision glaucoma surgery [1,2,3,4,5]. Their greatest contribution is not universal pressure-lowering potency, but anatomical specificity: they target the conventional outflow pathway while preserving conjunctival tissue and maintaining future filtration options [1,2,3,4,14,17,18,19,20]. By doing so, they enable earlier, safer, and more physiologically oriented intervention in appropriately selected eyes, particularly when moderate IOP reduction and medication reduction are sufficient therapeutic goals [17,18,19,20,27,28,29,30,31,32,33,34,35]. Their value is maximized when surgical selection is guided by glaucoma stage, target IOP, angle anatomy, and the likelihood that enhancement of conventional outflow will achieve the desired therapeutic objective [1,2,3,4,5].

## 5. Bleb-Forming and Conventional Filtering Surgery in the MIGS Era

The expansion of MIGS has reshaped the timing, indications, and philosophy of glaucoma surgery, but it has not reduced the clinical relevance of bleb-forming procedures or conventional filtering surgery [1,2,3,4,5]. Trabecular and Schlemm’s canal-based interventions have broadened the surgical spectrum by offering conjunctiva-sparing, physiologically oriented options for eyes requiring moderate IOP reduction [14,15,17,18,19,20]. However, their efficacy remains constrained by distal outflow resistance and episcleral venous pressure [1,2,3,4]. When optic nerve preservation requires substantially lower target IOP, particularly in advanced, refractory, or rapidly progressive glaucoma, procedures that bypass the conventional outflow system remain essential [4,6,7].

Bleb-forming surgery is based on a fundamentally different principle from trabecular and canal-based MIGS [4,21,22,23,26]. Instead of enhancing aqueous drainage through Schlemm’s canal and the distal collector system, these procedures establish an alternative pathway from the anterior chamber to the subconjunctival space [21,22,23,26]. This approach can achieve pressure levels that are often unattainable through conventional outflow enhancement alone [4,6,7]. The determinant of surgical success, however, shifts from canalicular and distal outflow physiology to conjunctival wound healing, bleb morphology, fibrosis modulation, postoperative inflammation, antimetabolite response, and long-term bleb surveillance [21,22,23,26]. Thus, the greater pressure-lowering potential of filtration surgery is inseparable from its biological dependence on the subconjunctival healing environment [21,22,23,26].

The XEN Gel Stent occupies an intermediate position within this surgical continuum and is best regarded as a minimally invasive bleb surgery (MIBS) procedure, representing a distinct filtration subgroup of MIGS [21,22,26]. Although commonly discussed within the MIGS spectrum because of its less invasive implantation profile, its mechanism is closer to filtration surgery than to angle-based trabecular bypass or Schlemm’s canal scaffolding [2,3,4,21,22,26]. By creating a controlled subconjunctival filtration route, XEN aims to combine a more standardized outflow pathway with a less invasive approach than traditional trabeculectomy [21,22,26]. Its potential advantage is greater IOP reduction than many conventional outflow MIGS procedures in selected eyes, whereas its limitations include subconjunctival fibrosis, bleb encapsulation, insufficient filtration, needling, antimetabolite modulation, and postoperative bleb management [21,22,23,26]. For this reason, XEN should be conceptualized as a minimally invasive filtration procedure rather than as a direct equivalent of trabecular or canal-based MIGS [2,3,4,21,26].

PreserFlo MicroShunt represents another subconjunctival filtration approach positioned between conventional MIGS and traditional trabeculectomy within the contemporary surgical continuum [2,3,4,21,22,23,26]. Similar to XEN implantation, its mechanism depends on the creation of an alternative subconjunctival drainage pathway rather than enhancement of conventional trabecular outflow [21,22,23,26]. However, PreserFlo is designed to provide controlled posterior filtration through an ab externo approach with the aim of reducing dependence on scleral dissection and iridectomy while maintaining substantial pressure-lowering efficacy [4,21,22,23]. Its role is particularly relevant in eyes requiring lower target IOP than is typically achievable with canal-based MIGS, but in which a less invasive alternative to trabeculectomy may be desirable [2,3,4,6,21,22]. Nevertheless, long-term success remains dependent on conjunctival wound healing, fibrosis modulation, bleb function, and postoperative management [23,26]. The procedure should therefore be regarded as MIBS rather than conventional outflow-enhancing MIGS procedure [2,3,4,21,22,23].

Trabeculectomy remains the reference procedure for achieving low IOP in many eyes with advanced glaucoma [4,6]. By creating a guarded fistula between the anterior chamber and the subconjunctival space, trabeculectomy bypasses the trabecular meshwork, Schlemm’s canal, collector channels, and episcleral venous limitations, thereby enabling pressure reduction beyond the physiological ceiling of conventional outflow surgery [4,6]. Evidence from advanced glaucoma populations continues to support its role as a highly effective intervention despite the growing availability of less invasive alternatives [6]. Nevertheless, this efficacy is accompanied by a substantial postoperative burden and a distinct complication profile, including hypotony, shallow anterior chamber, choroidal detachment, bleb leak, infection, scarring, bleb failure, and late bleb-related adverse events [4,6,8]. Rare but severe complications, including malignant glaucoma, further underscore the need for careful patient selection, meticulous surgical technique, and intensive postoperative monitoring [4,6,8].

Aqueous shunt implantation represents another indispensable component of contemporary glaucoma surgery, particularly in complex, refractory, secondary, or previously operated eyes [4,7]. Tube shunts create an alternative drainage route to an episcleral plate. Depending on the clinical setting, the tube may be inserted through the limbus, ciliary sulcus, or pars plana, with its distal tip positioned in the anterior chamber, ciliary sulcus, or vitreous cavity according to lens status, corneal endothelial risk, and the need for concomitant vitrectomy [4,7]. They are particularly valuable when conjunctival scarring, failed trabeculectomy, previous ocular surgery, or other risk factors reduce the predictability of standard filtration surgery [4,7]. Both valved and non-valved devices are widely used in contemporary practice. Valved implants may reduce the risk of early postoperative hypotony, whereas non-valved devices are often associated with greater long-term IOP reduction but typically require temporary flow-restricting measures during the early postoperative period [7]. Compared with trabecular and canal-based MIGS, aqueous shunts generally offer greater pressure-lowering potential but carry greater surgical and long-term risks, including corneal endothelial compromise, diplopia, tube exposure, erosion, motility disturbance, hypotony, plate encapsulation, and the need for revision surgery [4,7,13]. Selected combined approaches, such as adjunctive goniotomy with tube shunt implantation, have also been explored as strategies to address both proximal outflow resistance and alternative drainage pathways, although their evidence base remains preliminary [41].

The relationship between MIGS and conventional filtering surgery should therefore be understood as complementary rather than competitive [1,2,3,4,5]. MIGS has expanded the earlier and safer end of the surgical continuum, particularly for patients with mild-to-moderate glaucoma, visually significant cataract, medication intolerance, ocular surface disease, or adherence difficulties [2,3,4,5,36,37,38]. In contrast, XEN implantation, trabeculectomy, and aqueous shunt surgery remain necessary when the therapeutic objective requires more aggressive IOP reduction, especially in advanced, refractory, or rapidly progressive glaucoma [4,6,7,21,26]. Within a precision glaucoma surgery framework, the defining question is not whether a procedure is minimally invasive, but whether its mechanism, pressure-lowering capacity, safety profile, and postoperative demands are appropriate for the individual patient’s disease phenotype and target IOP [1,2,3,4,5,6,7,13].

## 6. Diagnostic Assessment of Outflow Pathways and Surgical Target Selection

A central challenge in precision glaucoma surgery is that the anatomical site of outflow resistance cannot yet be identified directly in routine clinical practice [1,2,3,4,5]. Nevertheless, preoperative evaluation increasingly extends beyond measurement of intraocular pressure alone and incorporates structural, anatomical, and functional information that may help guide selection of the most appropriate surgical pathway [2,3,4,5].

Gonioscopy remains the cornerstone of surgical planning because it determines angle accessibility and identifies anatomical features that influence the feasibility of trabecular and Schlemm’s canal-based procedures [1,2,3,4,5]. Anterior segment optical coherence tomography (AS-OCT) and ultrasound biomicroscopy further enhance anatomical assessment by providing objective characterization of angle configuration, anterior chamber depth, lens-related crowding, and iridocorneal relationships, particularly in eyes with angle-closure mechanisms or complex anterior segment anatomy [10,11].

Increasing attention has also been directed toward imaging and functional assessment of the conventional outflow system itself [35]. Visualization of Schlemm’s canal dimensions, collector channel architecture, episcleral venous fluid columns, and aqueous angiography patterns has improved understanding of the segmental nature of aqueous outflow and may eventually support more individualized targeting of canal-based procedures [35]. Although these technologies remain primarily investigational and are not yet routinely incorporated into clinical decision-making, they illustrate a future direction in which surgical planning may be guided by direct characterization of outflow pathway function rather than by indirect clinical surrogates alone [35].

Diagnostic evaluation may also assist in identifying circumstances in which enhancement of conventional outflow is less likely to achieve the desired pressure reduction [1,2,3,4,5]. Eyes with advanced disease requiring very low target intraocular pressure, extensive angle compromise, significant distal outflow dysfunction, or conjunctival pathology may be more appropriately managed with subconjunctival filtration procedures, aqueous shunt implantation, or alternative mechanism-based strategies [4,6,7]. Conversely, lens-related anatomical crowding identified by gonioscopy and anterior segment imaging may support lens extraction as a pathophysiologically targeted intervention [10,11].

Although no currently available diagnostic modality can definitively determine the optimal glaucoma procedure for an individual eye, advances in anterior segment imaging, aqueous outflow visualization, and functional pathway assessment are progressively moving glaucoma surgery toward a more personalized and mechanism-driven model of care [1,2,3,4,5,35,39].

## 7. Patient Selection and Proposed Mechanism-Based Surgical Algorithm

Appropriate patient selection is the central determinant of success in contemporary glaucoma surgery because the efficacy and safety of each procedure depend on the relationship between disease severity, anatomical target, required IOP reduction, and postoperative risk [1,2,3,4,5]. The expanding availability of MIGS has enabled earlier intervention with a more favorable safety profile in selected patients, but it has also increased the risk of oversimplified decision-making [1,2,3,4,5]. Procedures with different anatomical targets, pressure-lowering mechanisms, and postoperative requirements should not be regarded as interchangeable simply because they are less invasive than traditional filtration surgery [2,3,4,5,13]. Surgical planning should instead integrate glaucoma stage, baseline IOP, target IOP, rate of progression, angle anatomy, lens status, medication burden, ocular surface status, conjunctival condition, previous surgery, access to follow-up, and the likely site of aqueous outflow resistance [1,2,3,4,5,6,7,13,42]. The principal clinical determinants that should guide individualized surgical planning are summarized in Table 2.

The first step in surgical decision-making is to define the pressure level required to protect the optic nerve [1,2,3,4,5]. Glaucoma severity should be assessed according to the overall stage of structural and functional damage, based on the integration of optic nerve assessment, visual field loss, and the rate of disease progression rather than baseline IOP alone [1,2,3,4,5]. In eyes with mild-to-moderate glaucoma, the therapeutic objective may include meaningful IOP reduction, reduction in topical medication burden, improved tolerability, and preservation of future surgical options [1,2,3,4,5]. In this setting, trabecular and Schlemm’s canal-based procedures, including trabecular micro-bypass, canal scaffolding, goniotomy, trabeculotomy, and canaloplasty, may provide an appropriate balance between efficacy and safety [15,17,18,19,20,27,28,29,30,31,32,33,34,35]. In advanced glaucoma, however, severe structural damage or rapid visual field progression may require a low target IOP that is unlikely to be achieved reliably through conventional outflow enhancement alone [4,6]. In such eyes, trabeculectomy or aqueous shunt implantation should be considered early rather than after sequential failure of procedures with insufficient pressure-lowering potential [4,6,7].

Baseline IOP further modifies the expected benefit of each surgical pathway [27,42]. Eyes with higher preoperative IOP often demonstrate larger absolute pressure reductions, whereas eyes with lower baseline IOP generally achieve smaller reductions in absolute terms [27,42]. However, in normal-tension glaucoma, even modest IOP reductions may be clinically meaningful when they represent a substantial percentage decrease from baseline and contribute to slowing disease progression. In such patients, canal-based or trabecular procedures may still provide benefit through additional IOP lowering, medication reduction, or improved treatment tolerability, although individualized target IOP assessment remains essential [14,17,27,42]. Similarly, in combined cataract–glaucoma procedures, the IOP-lowering effect of phacoemulsification itself must be considered, particularly in eyes in which lens-related anatomy contributes to impaired aqueous dynamics [9,10,11,25,26,27,36,45]. Distinguishing the contribution of cataract extraction from the incremental effect of the glaucoma procedure is therefore essential when interpreting outcomes and counseling patients [9,25,26,27,38,45].

Medication burden and ocular surface disease represent additional indications for earlier surgical intervention [2,3,4,5]. Chronic topical therapy may be limited by preservative-related toxicity, conjunctival inflammation, adherence failure, cost, and reduced quality of life [2,3,4,5]. In patients with otherwise acceptable IOP control but substantial intolerance, poor adherence, or ocular surface compromise, conjunctiva-sparing MIGS may provide meaningful benefit by reducing treatment burden while maintaining adequate pressure control [1,2,3,4,5]. However, medication reduction should never supersede optic nerve protection. In progressive disease, reducing topical therapy is valuable only if postoperative IOP remains compatible with long-term preservation of visual function [2,3,4,5].

Lens status and angle anatomy should be assessed before any ab interno procedure [1,2,3,4,10,11]. Phacoemulsification combined with trabecular or canal-based MIGS is particularly suitable for patients with visually significant cataract and mild-to-moderate open-angle glaucoma [9,25,26,27,45]. In angle-closure disease, lens extraction may directly address the anatomical mechanism of IOP elevation by deepening the anterior chamber and widening the iridocorneal angle [10,11]. Additional angle surgery should be individualized according to gonioscopic visibility, extent of synechial closure, angle configuration, and procedural feasibility [10,11,43,44]. Poor visualization, extensive peripheral anterior synechiae, abnormal angle anatomy, or corneal opacity may increase the risk of malposition, incomplete treatment, or inadequate efficacy [43,44]. Reported complications such as Hydrus malposition, recurrent hyphema, inflammation, and uveitis–glaucoma–hyphema syndrome reinforce that MIGS requires precise technique, appropriate anatomical selection, and careful postoperative assessment [43,44].

Conjunctival status is equally important because glaucoma is a lifelong disease requiring long-term therapeutic sequencing [1,2,3,4,5]. One of the major advantages of trabecular and canal-based surgery is that it generally preserves the conjunctiva and avoids bleb formation, thereby maintaining future options for trabeculectomy or tube shunt implantation [1,2,3,4]. Subconjunctival microshunts and trabeculectomy may be more appropriate when lower IOP is required, but their success depends on bleb function, fibrosis modulation, antimetabolite response, and postoperative management [6,21,22,23,26]. Previous ocular surgery, failed blebs, tube implants, inflammation, and conjunctival scarring should therefore influence both procedure choice and surgical sequencing [4,7,21,22,23,26].

A practical mechanism-based surgical algorithm can be structured around the interaction between disease severity, target IOP, anatomical mechanism, and acceptable postoperative risk [1,2,3,4,5]. In mild-to-moderate open-angle glaucoma with visually significant cataract, medication burden, and a target IOP compatible with moderate pressure reduction, phacoemulsification combined with trabecular or Schlemm’s canal-based MIGS is often appropriate [25,26,27,36,37,38,45]. Segmental canal scaffolding may be considered when broader Schlemm’s canal engagement is desired; iStent-type procedures represent focal trabecular bypass; and implant-free approaches such as Trabectome, Kahook Dual Blade goniotomy, microhook trabeculotomy, or canaloplasty may be selected according to angle anatomy, surgeon experience, device availability, and desired extent of treatment [17,18,19,20,27,28,29,30,31,32,33,34,35]. When greater IOP reduction is required, subconjunctival microshunts, trabeculectomy, or aqueous shunts become more relevant [6,7,22,26]. When lens-induced crowding is the dominant mechanism, lens extraction may represent the most rational first intervention [10,11]. When reduction in aqueous production is clinically appropriate, cyclophotocoagulation-based procedures may be considered alone or as part of a combined strategy [12,25].

Patient-specific and healthcare system factors may also influence procedure selection and the feasibility of postoperative management [22,25,39,40]. Considerations such as ocular anatomy, scarring risk, access to follow-up, device availability, and local practice patterns should therefore be integrated into individualized surgical planning [39,40]. Studies in Asian eyes and analyses of changing global surgical trends emphasize that glaucoma surgery should be evaluated across diverse populations and healthcare systems rather than extrapolated from a single demographic or economic context [25,39,40]. In some settings, implant-free procedures such as goniotomy, trabeculotomy, canaloplasty, or cyclophotocoagulation may be more scalable than high-cost implant technologies [12,29,30,31,35,40].

The proposed algorithm should remain flexible rather than prescriptive, recognizing that the suitability of any procedure depends on disease phenotype, target IOP, progression rate, anatomical substrate, conjunctival reserve, and tolerance for postoperative risk [1,2,3,4,5,11,13]. The central principle is to define the pressure required to protect the optic nerve, identify the dominant anatomical mechanism, select the least morbid procedure capable of achieving the required target, preserve future options when clinically safe, and adapt the strategy to patient-specific and health-system realities [1,2,3,4,5,6,7]. The relative clinical characteristics of the principal surgical pathways are summarized in Table 3.

## 8. Illustrative Clinical Scenarios in Mechanism-Based Surgical Decision-Making

The principles of precision glaucoma surgery can be illustrated through representative clinical scenarios. In a patient with mild-to-moderate primary open-angle glaucoma, visually significant cataract, preserved angle anatomy, and a target intraocular pressure achievable through moderate pressure reduction, phacoemulsification combined with trabecular or Schlemm’s canal-based MIGS may provide an appropriate balance between efficacy, safety, medication reduction, and preservation of future surgical options [9,15,36,37,38].

In normal-tension glaucoma, surgical planning is driven primarily by the need for meaningful percentage-based IOP reduction despite a relatively low baseline IOP. Filtration procedures may therefore be considered earlier than in eyes with comparable structural damage but higher baseline pressures [4,6,7].

In contrast, a patient with advanced glaucoma and documented progression despite medical therapy may require substantially lower target intraocular pressure than can typically be achieved through conventional outflow enhancement alone. In such cases, subconjunctival filtration surgery, trabeculectomy, or aqueous shunt implantation may represent more appropriate strategies because the required degree of pressure reduction becomes the dominant determinant of surgical selection [4,6,7,21,22,23,26].

A different approach applies to angle-closure disease. When gonioscopy and anterior segment imaging demonstrate lens-related crowding and angle narrowing, lens extraction may directly address the underlying anatomical mechanism of intraocular pressure elevation and should therefore be considered a mechanism-based intervention rather than solely a visually rehabilitative procedure [10,11]. In pseudoexfoliation glaucoma, increased trabecular outflow resistance and potentially faster disease progression may justify earlier escalation to more extensive trabecular or canal-based procedures than would typically be considered in primary open-angle glaucoma with comparable IOP levels [1,2,3,4,5,13]. These examples illustrate how disease stage, glaucoma subtype, anatomical substrate, and target intraocular pressure collectively guide individualized surgical planning within a precision glaucoma surgery framework [1,2,3,4,5,13].

## 9. Safety, Failure Mechanisms, and Evidence Standards

The safety profile of contemporary glaucoma surgery should be interpreted in relation to anatomical target, mechanism of action, disease severity, and the expected duration of benefit [1,2,3,4,5,13]. MIGS has expanded the possibility of earlier surgical intervention by reducing perioperative morbidity compared with conventional filtration surgery, but minimal invasiveness should not be equated with absence of risk [1,2,3,4,5,13]. Rather, each surgical pathway shifts risk toward a different anatomical compartment. Trabecular and Schlemm’s canal-based procedures are primarily vulnerable to angle-related complications and distal outflow limitation; subconjunctival procedures depend on bleb biology and wound-healing modulation; suprachoroidal or supraciliary procedures raise specific concerns regarding anterior segment and endothelial safety; and ciliary body-directed interventions require careful titration of aqueous suppression [1,2,3,4,5,21,22,23,25,26].

Early postoperative events after angle-based surgery are generally less severe than those associated with trabeculectomy or tube shunt implantation, but they remain clinically relevant [15,17,18,19,20,27,28,29,30,31,32,33,34,35]. Transient hyphema, mild inflammation, short-term IOP fluctuation, peripheral anterior synechiae, and corneal edema may occur after trabecular micro-bypass, Schlemm’s canal scaffolding, goniotomy, trabeculotomy, or canaloplasty [17,18,19,20,27,28,29,30,31,32,33,34,35]. Some of these findings reflect the intended surgical mechanism, particularly direct communication between the anterior chamber and Schlemm’s canal after trabecular disruption [18,19,20,28,29,30,31]. However, persistent hyphema, recurrent inflammation, device obstruction, sustained IOP elevation, or progressive synechial closure should prompt evaluation for malposition, impaired patency, inadequate collector channel recruitment, or distal outflow failure [1,2,3,4,43,44].

Device-based MIGS introduces additional safety considerations related to implant position, patency, tissue interaction, and long-term anatomical stability [32,33,34,43,44]. In Schlemm’s canal-based implant surgery, accurate intracanalicular placement is essential because the device must bypass the trabecular meshwork while remaining appropriately seated within the canal [36,37,38,43,44]. Randomized trials, systematic review evidence, and long-term HORIZON data support an acceptable safety profile for Hydrus in appropriately selected patients, particularly when implanted in combination with cataract surgery [15,17,36,37,38]. Nevertheless, rare complications, including malposition, recurrent hyphema, inflammation, uveitis–glaucoma–hyphema syndrome, elevated IOP, and device removal, have been reported [43,44]. These events do not negate the overall evidence supporting canal-based MIGS, but they emphasize that such procedures require precise implantation, postoperative gonioscopy, and long-term anatomical surveillance [13,43,44].

Corneal endothelial safety has become a critical endpoint in the evaluation of MIGS and related anterior segment implants [13,16]. Endothelial risk cannot be inferred solely from incision size, early postoperative recovery, or the absence of a bleb. It depends on implant location, anterior chamber configuration, proximity to the corneal endothelium, inflammatory response, device stability, and long-term tissue interaction [13,43,44]. This issue has been particularly instructive in the context of supraciliary procedures, but endothelial monitoring is also relevant for angle-based implants and tube shunts, especially in eyes with shallow anterior chambers, abnormal angle anatomy, previous surgery, or pre-existing endothelial compromise [7,13,16,43,44].

Bleb-forming procedures introduce a distinct spectrum of complications because their success depends on the creation and long-term maintenance of subconjunctival filtration [4,6,21,22,23,26]. XEN implantation, trabeculectomy, and related filtration procedures can achieve lower IOP than many canal-based operations, but their outcomes are strongly influenced by bleb morphology, conjunctival wound healing, fibrosis modulation, and postoperative management [6,21,22,23,26]. Fibrosis, bleb encapsulation, insufficient filtration, hypotony, bleb leak, blebitis, endophthalmitis, choroidal complications, and late bleb failure remain central safety concerns [4,21,22,23,26]. Rare but severe events, including malignant glaucoma after trabeculectomy, further illustrate the importance of careful patient selection, early recognition of complications, and structured postoperative follow-up [4,6,8]. Aqueous shunts add further risks related to corneal endothelial damage, tube exposure, motility disturbance, diplopia, plate encapsulation, hypotony, and revision surgery [4,7,13].

Failure mechanisms should be interpreted according to the surgical target rather than described generically as surgical failure [1,2,3,4,5]. Trabecular and canal-based procedures may fail because of distal outflow resistance, peripheral anterior synechiae, device obstruction, inadequate access to functional collector channels, or mismatch between the pressure-lowering capacity of the procedure and the required target IOP [35,43,44]. Bleb-forming procedures may fail because of subconjunctival fibrosis, bleb encapsulation, inflammation, or insufficient modulation of wound healing [4,6,21,22,23]. Tube shunts may fail because of plate encapsulation, tube obstruction, exposure, or anatomical complications [4,7]. Cyclophotocoagulation-based procedures require a different balance: sufficient reduction in aqueous production must be achieved without excessive inflammation, hypotony, or loss of ciliary body function [12,25]. Thus, failure should not be understood only as technical failure; it may also reflect incorrect matching between the surgical mechanism and the dominant pathophysiological driver of disease [1,2,3,4,5].

The rapid adoption of MIGS has also highlighted important limitations in the evidence base [2,3,5]. Several procedures entered clinical practice with relatively short follow-up, heterogeneous endpoints, variable medication washout protocols, inconsistent definitions of success, and limited head-to-head comparative data [2,3,5]. These limitations are particularly relevant in glaucoma, where the value of a surgical intervention is determined not only by early postoperative IOP reduction, but also by durability, visual field stability, structural preservation, cumulative complication risk, need for reoperation, medication burden, quality of life, cost-effectiveness, and the ability to preserve future surgical options [2,3,4,5,13]. Future studies should therefore prioritize standardized endpoints, longer follow-up, transparent reporting of baseline IOP and medication status, endothelial cell density monitoring, postoperative intervention burden, and clinically meaningful definitions of success [2,3,4,5,13]. An overview of the current evidence landscape across the principal glaucoma surgical pathways, including the predominant study designs, follow-up maturity, and common methodological limitations, is provided in Table 4.

Long-term surveillance should be considered an integral component of surgical success [4,7,13]. After angle-based procedures, gonioscopy remains essential to assess device position, patency, peripheral anterior synechiae, pigment obstruction, and angle anatomy [43,44]. After bleb-forming procedures, follow-up must focus on bleb morphology, leakage, fibrosis, infection risk, hypotony, and late failure [21,22,23,26]. After tube shunt surgery, monitoring should include tube position, endothelial status, plate encapsulation, motility symptoms, and exposure risk [4,7,13]. The future of glaucoma surgery will depend not only on the development of new procedures, but also on defining how their benefits, limitations, and cumulative risks evolve over the patient’s lifetime [2,3,4,5,13].

## 10. Future Directions: Toward Precision Glaucoma Surgery

The next stage in glaucoma surgery is unlikely to be defined solely by the introduction of additional devices. The more important transition will be conceptual: from procedure-centered surgery toward precision, mechanism-based intervention [1,2,3,4,5]. In this model, the surgical objective is not simply to lower IOP, but to identify the dominant anatomical and physiological mechanism responsible for insufficient pressure control and to select an intervention capable of achieving the required target IOP with acceptable durability, safety, and long-term therapeutic flexibility [1,2,3,4,5,13].

Future surgical planning will increasingly depend on improved anatomical and functional assessment of aqueous humor pathways [1,2,3,4,5,35]. Current decision-making relies primarily on gonioscopy, IOP level, disease stage, medication burden, lens status, and surgeon experience [2,3,4,5]. Although indispensable, these parameters provide only indirect information about Schlemm’s canal, collector channels, episcleral venous drainage, and regional outflow capacity [1,2,3,4,35]. This limitation is particularly relevant for canal-based procedures, because trabecular micro-bypass, segmental Schlemm’s canal scaffolding, trabecular ablation, excisional goniotomy, ab interno trabeculotomy, and canaloplasty all depend on functional communication between the treated trabecular–canalicular segment and the distal outflow system [17,18,19,20,27,28,29,30,31,32,33,34,35]. More precise assessment of regional outflow activity may eventually allow surgeons to individualize not only the choice of procedure, but also the location, extent, and sequencing of treatment [2,3,4,5,35].

Potential predictive biomarkers may further refine individualized surgical planning. The most promising candidates include anatomical and imaging-based parameters such as angle configuration, Schlemm’s canal morphology, collector channel architecture, and lens-related anatomical features identified by gonioscopy and anterior segment imaging [10,11,35]. Corneal endothelial status and ocular biomechanical characteristics may also influence procedure selection in selected patients [7,13,24]. Although no validated biomarker currently provides reliable procedure-specific prediction, advances in outflow pathway characterization may support more personalized glaucoma surgery in the future [1,2,3,4,5,35].

A central unresolved question is how much of the conventional outflow pathway should be modified to achieve durable pressure reduction [30,31,35]. Focal trabecular bypass procedures are tissue-sparing and technically efficient, but their effect may be limited if the treated area does not correspond to a functional collector channel region [14,27,34]. Broader interventions, including segmental canal scaffolding, ab interno trabeculotomy, excisional goniotomy, and canaloplasty, may increase the likelihood of engaging functional distal pathways, but may also involve greater manipulation of the trabecular–canalicular system [30,31,32,33,35,36,37,38]. Future studies should examine how the extent of anatomical treatment interacts with baseline IOP, distal outflow function, glaucoma subtype, and long-term outcomes [2,3,4,5,30,31,32,33,34,35].

Cataract surgery will remain a major platform for glaucoma intervention [9,36,37,38,45]. In open-angle glaucoma, phacoemulsification provides an opportunity to combine lens removal with trabecular or canal-based MIGS, particularly in patients with mild-to-moderate disease and visually significant cataract [15,25,26,27,36,37,38]. In angle-closure glaucoma, lens extraction may directly modify the anatomical mechanism of disease by deepening the anterior chamber and widening the iridocorneal angle [10,11]. Comparative studies evaluating phacoemulsification combined with different glaucoma procedures, including trabecular micro-bypass, segmental canal scaffolding, nonpenetrating deep sclerectomy, XEN implantation, goniotomy, and micropulse transscleral laser therapy, illustrate the need to interpret combined surgery according to both lens status and glaucoma mechanism rather than as a uniform category of cataract-plus-glaucoma intervention [25,26,27,28,45].

Combination and sequential strategies are also likely to become increasingly important as glaucoma care becomes more individualized [2,3,4,5,41]. Selected approaches combining cataract extraction, excisional goniotomy, and transscleral cyclophotocoagulation illustrate the possibility of addressing lens-related anatomy, trabecular resistance, and aqueous production within a single therapeutic plan [12]. Similarly, adjunctive goniotomy combined with aqueous shunt implantation reflects interest in targeting both proximal outflow resistance and alternative drainage pathways in complex cases [41]. Combination procedures should be reserved for eyes in which additive benefit is clinically justified and supported by an acceptable safety profile [2,3,4,5,13,41].

The next generation of glaucoma surgery will also depend on biomaterials, implant design, endothelial safety, and drug–device integration [2,3,4,5,13]. Implant-based procedures must demonstrate long-term anatomical stability, endothelial safety, and predictable performance across diverse anterior segment anatomies [13,16,43,44]. Future innovation in subconjunctival devices should focus on improving flow control and wound-healing modulation while reducing postoperative complications and intervention burden [4,21,22,23,26]. Integration of surgery with sustained drug delivery may also become relevant, particularly in patients with poor adherence, ocular surface disease, medication intolerance, or increased risk of conjunctival scarring [2,3,4,5].

Predictive modeling may further support individualized surgical planning [2,3,4,5,35]. Future algorithms could incorporate baseline IOP, angle anatomy, lens status, corneal parameters, glaucoma stage, visual field progression, medication history, ocular surface status, conjunctival condition, prior surgery, ethnicity, scarring tendency, and patient-specific tolerance for postoperative burden [11,13,35,42]. Such tools could help estimate the likelihood of success for trabecular bypass, Schlemm’s canal scaffolding, goniotomy, trabeculotomy, canaloplasty, XEN implantation, trabeculectomy, tube shunt surgery, cyclophotocoagulation, or lens extraction [14,15,16,17,18,19,20,21,22,23,24,25,26,27,28,29,30,31,35]. Predictive models should complement clinical judgment and require external validation before widespread implementation [2,3,4,5].

Real-world evidence will be essential for translating precision glaucoma surgery into routine clinical practice [39,40]. Device availability, healthcare resources, and postoperative follow-up capacity will continue to influence the implementation of precision glaucoma surgery across different practice settings [39,40]. Therefore, progress cannot depend exclusively on high-cost implant technologies. Implant-free procedures such as goniotomy, trabeculotomy, canaloplasty-based surgery, and cyclophotocoagulation may remain particularly important in settings where device access is limited or affordability strongly influences care [12,29,30,31,35,39,40]. A globally applicable surgical framework should prioritize adaptability, mechanism-based reasoning, and health-system feasibility rather than a single device-centered model [2,3,4,5,39,40].

Within this future framework, canal-based MIGS will remain relevant not because any single device is universally applicable, but because these procedures exemplify a broader surgical concept: restoration or enhancement of conventional outflow through anatomically targeted modification of the trabecular meshwork and Schlemm’s canal [17,18,19,20,27,28,29,30,31,32,33,34,35]. Their optimal role will remain specific to eyes in which moderate IOP reduction, medication reduction, conjunctival preservation, and favorable safety are clinically meaningful, particularly when distal outflow pathways are sufficiently functional [13,14,15,27,28,29,30,31,32,33,34,35]. Future research should clarify which anatomical phenotypes derive the greatest benefit from focal bypass, segmental canal scaffolding, trabecular excision, trabeculotomy, or canaloplasty, and whether preoperative or intraoperative assessment can identify eyes most likely to respond [2,3,4,5,30,31,32,33,34,35].

The future of glaucoma surgery will therefore depend on individualized, mechanism-guided procedure selection rather than minimal invasiveness alone [1,2,3,4,5]. MIGS has broadened the available surgical strategies while supporting earlier and safer intervention in appropriately selected patients [1,2,3,4,5]. Its greatest long-term contribution may be the shift from standardized surgical escalation toward individualized therapeutic sequencing, in which anatomical assessment, expected pressure-lowering capacity, safety profile, and future surgical flexibility are integrated before intervention [1,2,3,4,5,13].

## 11. Conclusions

Glaucoma surgery is entering an era in which procedure selection should be guided by anatomical mechanism, disease phenotype, target IOP, and long-term therapeutic strategy rather than by invasiveness, device category, or historical treatment sequence alone. MIGS has expanded the surgical continuum and enabled earlier intervention, but its role is best understood within a broader framework that includes conventional outflow enhancement, subconjunctival filtration, trabeculectomy, aqueous shunts, lens-based anatomical modulation, and ciliary body-directed treatments.

Trabecular and Schlemm’s canal-based procedures are most appropriate when enhancement of conventional outflow is likely to achieve the required target IOP with an acceptable safety profile. Their principal advantages are conjunctiva preservation, physiological outflow enhancement, and reduction in medication burden in appropriately selected eyes. However, their efficacy remains constrained by distal outflow resistance and episcleral venous pressure and should not be expected to replace filtration or tube surgery when very low target IOP is required.

The future of glaucoma surgery will depend on matching each procedure to the patient’s disease stage, anatomical target, required pressure reduction, medication burden, safety profile, conjunctival status, and long-term therapeutic strategy. Continued advances in imaging, biomaterials, predictive modeling, and high-quality comparative evidence will further support individualized surgical planning. Ultimately, the key question is not which procedure is universally superior, but which mechanism is most appropriate for a given eye at a specific stage of disease to preserve visual function throughout the patient’s lifetime.

## Figures and Tables

**Table 1:** Mechanism-based classification of contemporary glaucoma surgery according to anatomical target and pressure-lowering pathway.

Anatomical Target and Pressure-Lowering Pathway	Primary Anatomical Target	Mechanism of IOP Reduction	Representative Procedures	Most Appropriate Clinical Role	Key Limitations	Surgical Access
Trabecular micro-bypass	Trabecular meshwork and Schlemm’s canal	Bypasses trabecular resistance and improves access to conventional outflow	iStent, iStent inject	Mild-to-moderate open-angle glaucoma, often combined with phacoemulsification	Limited by distal outflow resistance and episcleral venous pressure	Ab interno
Schlemm’s canal scaffolding	Schlemm’s canal and collector channel access	Maintains canal patency over a segment and enhances conventional outflow	Hydrus Microstent	Mild-to-moderate open-angle glaucoma, especially with cataract surgery	Requires functional distal outflow; not intended for very low target IOP	Ab interno
Trabecular excision/ablation/trabeculotomy	Trabecular meshwork and inner wall of Schlemm’s canal	Removes or incises trabecular resistance over a defined extent	Trabectome, Kahook Dual Blade, microhook trabeculotomy, Suture trabeculotomy (GATT)	Implant-free conventional outflow enhancement	Hyphema, variable effect depending on distal outflow function	Predominantly ab interno *
Canaloplasty-based procedures	Schlemm’s canal and distal conventional outflow system	Dilates Schlemm’s canal and may improve access to collector channels	Canaloplasty, STREAMLINE canaloplasty	Broader canal-based outflow restoration	Efficacy depends on distal pathway integrity	Ab interno or ab externo
Suprachoroidal/supraciliary procedures	Suprachoroidal or supraciliary space	Enhances uveoscleral outflow	Suprachoroidal microstent approaches	Alternative non-bleb outflow pathway	Long-term safety and endothelial concerns	Ab interno
Subconjunctival filtration	Subconjunctival space	Creates an alternative filtration route	XEN, Preserflo MicroShunt, trabeculectomy	Greater IOP reduction when canal-based surgery is insufficient	Fibrosis, bleb-related complications, postoperative interventions	Ab interno (XEN) or ab externo (PreserFlo, trabeculectomy)
Aqueous shunt surgery	Anterior chamber to episcleral plate	Diverts aqueous to an external reservoir	Tube shunts	Advanced, refractory, or previously operated glaucoma	Tube-related complications, endothelial risk, revision burden	Ab externo
Ciliary body-directed procedures	Ciliary body	Reduces aqueous production	Endoscopic cyclophotocoagulation (ECP); conventional transscleral cyclophotocoagulation (CW-TSCPC); micropulse transscleral cyclophotocoagulation (MP-TSCPC)	Selected eyes or combined strategies	Requires balance between efficacy and excessive aqueous suppression	Non-incisional transscleral or minimally invasive intraocular
Lens-based anatomical modulation	Lens and anterior chamber angle	Deepens anterior chamber and widens angle	Cataract extraction, clear-lens extraction	Angle-closure disease; combined platform in open-angle glaucoma	Effect depends on lens-related anatomy and glaucoma subtype	Ab interno

* Depending on the specific surgical technique. Abbreviations: CW-TSCPC—conventional transscleral cyclophotocoagulation; ECP—endoscopic cyclophotocoagulation; GATT—gonioscopy-assisted transluminal trabeculotomy; IOP—intraocular pressure; MP-TSCPC—micropulse transscleral cyclophotocoagulation; XEN—XEN Gel Stent.

**Table 2:** Key clinical determinants of individualized surgical planning in glaucoma.

Determinant	Clinical Relevance	Implication for Surgical Planning	References
Glaucoma stage and rate of progression	Determines the urgency and magnitude of required IOP reduction	Advanced or rapidly progressive disease may require procedures capable of achieving lower target IOP	[4,6,7]
Target IOP	Defines whether moderate or substantial pressure reduction is needed	Canal-based MIGS is most suitable when moderate IOP reduction is sufficient; filtration or tube surgery may be required for very low targets	[1,2,3,4,6,7]
Baseline IOP	Influences both the absolute and relative magnitude of postoperative IOP reduction	Lower baseline IOP, including normal-tension glaucoma, may limit the achievable absolute IOP reduction; however, even modest reductions may be clinically meaningful when they result in an appropriate percentage decrease from baseline	[42]
Angle anatomy	Determines feasibility and safety of ab interno procedures	Poor visualization, synechial closure, or abnormal angle configuration may reduce suitability for angle-based MIGS	[10,11,43,44]
Lens status	Influences anterior segment anatomy and the opportunity for combined surgery	Cataract extraction may be combined with MIGS in selected open-angle glaucoma or used as a mechanism-directed intervention in angle closure	[9,10,11,15,27,36,37,38]
Medication burden and ocular surface status	Affects tolerability, adherence, and quality of life	Conjunctiva-sparing procedures may be considered when reducing topical therapy is a major goal	[2,3,4,5]
Conjunctival condition	Determines future feasibility of bleb-forming or tube-based surgery	Preserving conjunctiva may be important in earlier disease; scarring or previous surgery may favor alternative approaches	[4,7,21,22,23,26]
Previous glaucoma surgery	Modifies anatomical access, scarring risk, and likelihood of success	Failed filtration surgery or prior implants may influence sequencing toward tube shunts or individualized combined approaches	[4,7,41]
Corneal endothelial status	Relevant for long-term safety of anterior segment implants and tube surgery	Eyes with endothelial vulnerability require careful procedure selection and postoperative monitoring	[7,13]
Healthcare context and follow-up capacity	Influences feasibility, cost, and postoperative management	Device availability, surgeon experience, reimbursement, and access to monitoring should inform surgical choice	[12,29,30,31,35,39,40]

Abbreviations: IOP—intraocular pressure; MIGS—microinvasive glaucoma surgery.

**Table 3:** Comparative clinical characteristics of major glaucoma surgical pathways.

Surgical Approach	Typical IOP-Lowering Capacity	Medication Reduction	Conjunctival Preservation	Postoperative Burden	Most Appropriate Clinical Role
Trabecular and Schlemm’s canal-based MIGS (iStent, Hydrus, goniotomy, trabeculotomy, canaloplasty)	Moderate	Moderate	Yes	Low	Mild-to-moderate open-angle glaucoma, often combined with cataract surgery
Subconjunctival microshunts (XEN, PreserFlo)	Moderate-to-substantial	Moderate-to-high	No	Moderate	Eyes requiring greater IOP reduction than typically achievable with canal-based MIGS
Trabeculectomy	Substantial	High	No	High	Advanced or rapidly progressive glaucoma requiring low target IOP
Aqueous shunt surgery	Substantial	High	No	High	Refractory, secondary, or previously operated glaucoma
Ciliary body-directed procedures	Variable	Variable	Yes	Low-to-moderate	Selected eyes or adjunctive treatment strategies
Lens-based anatomical intervention	Mild-to-moderate	Variable	Yes	Low	Angle-closure disease and selected combined cataract–glaucoma procedures

Abbreviations: IOP—intraocular pressure; MIGS—microinvasive glaucoma surgery.

**Table 4:** Overview of the current evidence base for major glaucoma surgical pathways.

Surgical Pathway	Predominant Evidence Sources	Follow-Up Maturity	Common Limitations of Current Evidence
Trabecular micro-bypass (iStent)	Randomized controlled trials, Cochrane reviews, systematic reviews, long-term prospective studies	Moderate-to-long term	Many studies include combined cataract surgery and selected mild-to-moderate POAG populations
Schlemm’s canal scaffolding (Hydrus Microstent)	Randomized controlled trials, Cochrane reviews, network meta-analyses, long-term extension studies	Long term	Evidence largely derived from selected open-angle glaucoma populations undergoing phacoemulsification
Trabecular excision, goniotomy, and trabeculotomy	Prospective comparative studies, retrospective series, systematic reviews	Moderate term	Heterogeneous surgical techniques, outcome measures, and study designs
Canaloplasty-based procedures	Prospective studies, comparative studies, emerging randomized evidence	Moderate term	Smaller evidence base and variability in procedural approaches
Suprachoroidal procedures	Cochrane review and device-specific clinical studies	Limited-to-moderate	Limited long-term data and heterogeneous clinical experience
Subconjunctival microshunts (XEN Gel Stent, PreserFlo MicroShunt)	Prospective studies, comparative cohorts, real-world studies, systematic reviews	Moderate term	Variability in antimetabolite use, needling rates, bleb management, and success definitions
Trabeculectomy	Randomized trials, long-term comparative studies, systematic reviews	Long term	Technique-dependent outcomes and variability in postoperative management
Aqueous shunt surgery	Cochrane review, comparative studies, long-term clinical experience	Long term	Device heterogeneity and differences between valved and non-valved implants
Cyclophotocoagulation-based procedures	Prospective studies, retrospective series, comparative studies	Moderate term	Variable treatment protocols and outcome definitions

Abbreviations: POAG—primary open-angle glaucoma; RCT—randomized controlled trial; IOP—intraocular pressure.

## Data Availability

No new data were created or analyzed in this study. Data sharing is not applicable to this article.

## Abbreviations

The following abbreviations are used in this manuscript:

AS-OCT	Anterior segment optical coherence tomography
CW-TSCPC	Continuous-wave transscleral cyclophotocoagulation
ECP	Endoscopic cyclophotocoagulation
GATT	Gonioscopy-assisted transluminal trabeculotomy
IOP	Intraocular pressure
MIBS	Minimally invasive bleb surgery
MIGS	Microinvasive glaucoma surgery
MP-TSCPC	Micropulse transscleral cyclophotocoagulation
POAG	Primary open-angle glaucoma
XEN	XEN Gel Stent
